# Role of Ultraviolet Radiation in Papillomavirus-Induced Disease

**DOI:** 10.1371/journal.ppat.1005664

**Published:** 2016-05-31

**Authors:** Aayushi Uberoi, Satoshi Yoshida, Ian H. Frazer, Henry C. Pitot, Paul F. Lambert

**Affiliations:** 1 McArdle Laboratory for Cancer Research, Department of Oncology, School of Medicine and Public Health, University of Wisconsin-Madison, Madison, Wisconsin, United States of America; 2 The University of Queensland Diamantina Institute, Translational Research Institute, Brisbane, Queensland, Australia; Fred Hutchinson Cancer Research Center, UNITED STATES

## Abstract

Human papillomaviruses are causally associated with 5% of human cancers. The recent discovery of a papillomavirus (MmuPV1) that infects laboratory mice provides unique opportunities to study the life cycle and pathogenesis of papillomaviruses in the context of a genetically manipulatable host organism. To date, MmuPV1-induced disease has been found largely to be restricted to severely immunodeficient strains of mice. In this study, we report that ultraviolet radiation (UVR), specifically UVB spectra, causes wild-type strains of mice to become highly susceptible to MmuPV1-induced disease. MmuPV1-infected mice treated with UVB develop warts that progress to squamous cell carcinoma. Our studies further indicate that UVB induces systemic immunosuppression in mice that correlates with susceptibility to MmuPV1-associated disease. These findings provide new insight into how MmuPV1 can be used to study the life cycle of papillomaviruses and their role in carcinogenesis, the role of host immunity in controlling papillomavirus-associated pathogenesis, and a basis for understanding in part the role of UVR in promoting HPV infection in humans.

## Author Summary

Epidemiological studies have implicated that ultraviolet radiation (UVR) from sunlight drive papillomavirus-induced disease in healthy as well as immunocompromised humans. In this report we demonstrate that treatment of immunocompetent mice with UVR renders them susceptible to papillomas and associated squamous cell carcinoma when infected with the recently discovered murine papillomavirus (MmuPV1). Our data further suggest UVR increases susceptibility to virally induced disease by inducing immunosuppression.

## Introduction

Papillomaviruses are species-specific, epitheliotropic, double-stranded DNA viruses. There are over 200 strains or genotypes of human papillomaviruses (HPVs) [1]. Mucosotropic HPVs are the most common sexually transmitted pathogens, and a subset of these viruses cause 5% of human cancers, including cervical cancer, other anogenital cancers, and a growing fraction of head and neck cancers (reviewed in [2]). Other HPVs cause cutaneous warts, which are among the most common ailments treated by dermatologists [3–6]. They arise most frequently among children [7,8], and impose a significant burden in immunocompromised patients, particularly amongst organ transplant recipients [9–11]. They are ubiquitous in nature and can persist in the skin asymptomatically for years most clearly in context of immunosuppressed patients [9,12]. A subset of cutaneous HPVs also has been causally associated with skin cancer (reviewed in [9,13,14]).

The study of papillomavirus-induced disease has long been hindered by the absence of any identified strains of virus that infect laboratory mice. This limitation was overcome with the recent identification of the murine papillomavirus, MmuPV1, isolated from cutaneous warts arising on the T-cell deficient *NMRI-FoxN1* ^*nu/nu*^ strain of laboratory mice [15]. MmuPV1 belongs to the *pi*-papillomaviridae genus and is phylogenetically related to cutaneous HPVs and other animal PVs that cause cutaneous disease in exotic rodent species [16]. MmuPV1 causes warts in cutaneous epithelium as well as in mucosal epithelium lining the female reproductive tract and oral cavity [15,17–20], and in some cases these lesions show signs of neoplastic progression [20,21]. Multiple studies have shown that the ability of MmuPV1 to cause overt disease is largely restricted to immunodeficient strains of mice [18,20].

Epidemiological studies have suggested that there is a correlation between exposure to ultraviolet radiation (UVR) and the prevalence of cutaneous HPVs in healthy and immunosuppressed patients, respectively [22,23]. Cutaneous HPVs are more commonly found at anatomical sites exposed to sunlight, and a history of blistering sunburn is associated with prevalent and persistent cutaneous HPV infections [12,22–24]. UVR has also been shown to play a role in papillomavirus-associated disease caused by animal papillomaviruses that infect the African multimammate rat (MnPV) or the cottontail rabbit (CRPV) [25–27].

In this report we demonstrate that UVR, specifically UVB (280 to 315 nm) assists in development of MmuPV1 dependent papillomas and associated malignant progression to squamous cell carcinomas, in immunocompetent strains of mice. We further show that there is a correlation between UVR-induced susceptibility to MmuPV-1 associated disease and UVR-induced immunosuppression. These findings provide a potential explanation for the role of UVR-mediated immunosuppression in papillomavirus-associated disease in humans.

## Results

### MmuPV1 causes papillomas in immunocompetent FVB mice following exposure to UV irradiation

Several lines of evidence support a link between UVR exposure and papillomavirus infection [22,23,26]. We sought to determine if UVR facilitates MmuPV1-induced papillomatosis in immunocompetent strains of mice. We used the inbred FVB/NJ strain of mice for our initial studies because it has been classically used to study chemically induced skin tumorigenesis [28].

**Fig 1:**
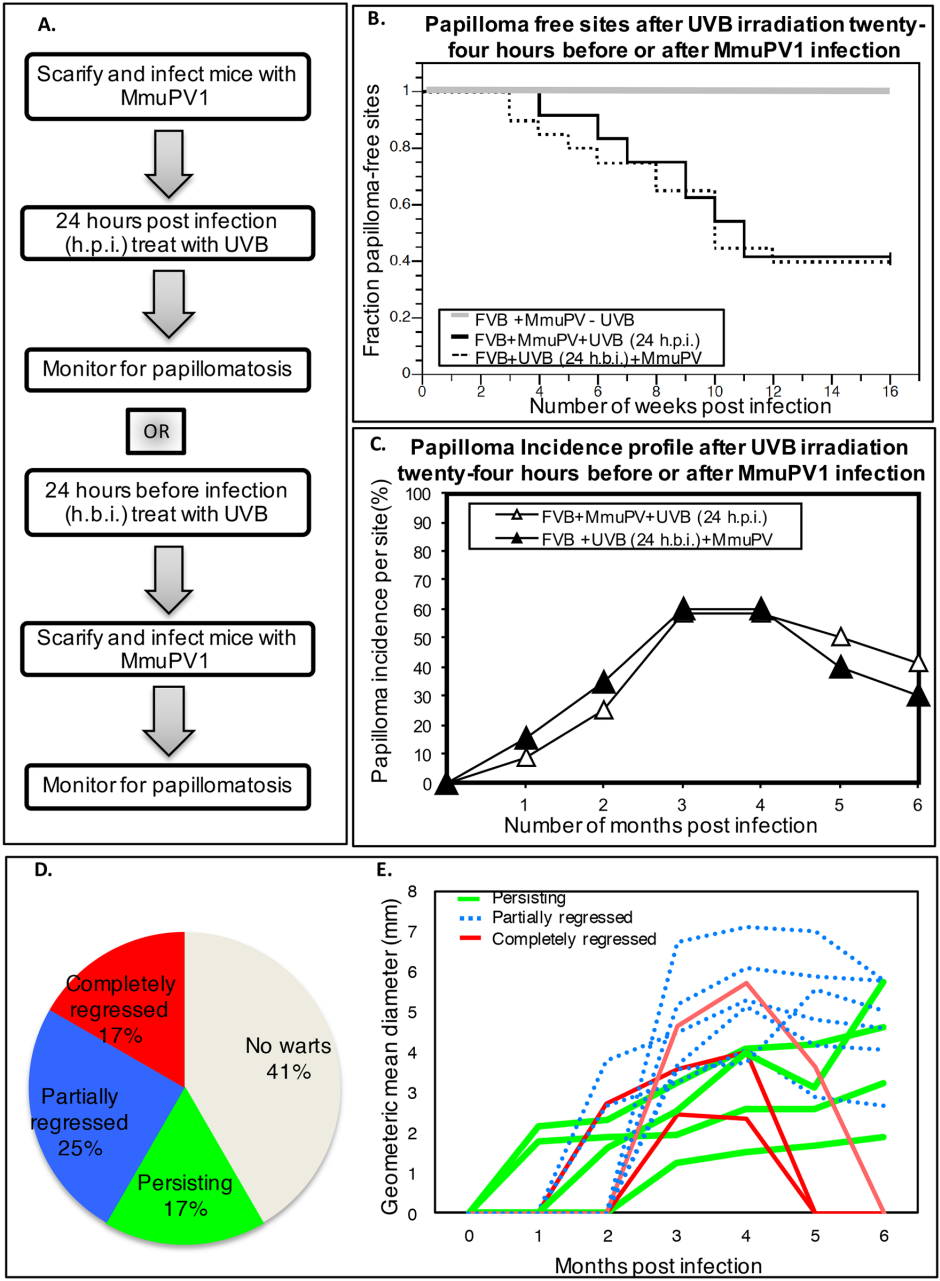
MmuPV1-induced papillomas in UVB-irradiated FVB/NJ mice. **(A)** Sequence of manipulations to FVB/NJ mice in the MmuPV1-UVB infection model system. Ear sites were scarified and exposed to 10^8^ VGE of MmuPV1 either 24 hours before (FVB+UVB 24h.b.i.+MmuPV) or 24 hours after (FVB+MmuPV+UVB 24h.p.i.) 300mJ/cm^2^ UVB whole-body irradiation. Sites were then scored weekly for presence of papillomas. **(B)** Kaplan-Meyer plots of the fraction of papilloma-free infected ear sites with respect to time. There is no significant difference in the temporal onset of disease between the two experimental groups (FVB+UVB24h.p.i.+MmuPV vs FVB+MmuPV+UVB 24h.b.i.; *P* = 0.814, Wilcoxon log-rank test, *two-sided*). **(C)** Percentage of sites with overt papillomas over a 6-month observation period. In both the cohorts of mice, a subset of papillomas completely regressed by 6 months post-infection. (D) Distribution of ear sites of UVB-treated FVBN/J mice (FVB+UVB24h.p.i.+MmuPV group) infected that developed papillomas that completely regressed (red), partially regressed (blue) or continued to grow (green) over the 6 month monitoring period. In grey is the fraction of sites that did not develop papillomas. (E) Growth profiles of individual papillomas arising in the FVB+UVB24h.p.i.+MmuPV group during the 6-month monitoring period. Individual lines represent the geometric mean diameter of each papilloma as a function of time. Red lines are papillomas that completed regressed. Blue lines are papillomas that partially regressed. Green lines are papillomas that continued to grow.

Ears and tails of 8–10 weeks old FVB/NJ mice were infected with 10^8^ viral genome equivalents (VGE, a measure of the amount of encapsidated genomes in a stock of virus) of MmuPV1 virions following topical scarification of the epidermis. Twenty-four hours post-infection (h.p.i.), mice were or were not exposed to varying doses of UVB whole body irradiation (280-320nm range UVR). Mice were then observed weekly for papillomatosis (Fig 1A). By 3 months post-infection greater than 50% of infected ear sites developed papillomas in FVB/NJ mice treated with 300mJ/cm^2^ UVB (Fig 1B, Table 1, S1 Fig-Panel A). Sites on the tails of the same mice infected with an equal dose of virus failed to develop papillomas. We did not see any papillomas develop at infected sites on non-irradiated mice or mice treated with a lower dose (150mJ/cm^2^) of UVB (Table 1). Mock-infected mice exposed to the same doses of UVB did not develop warts (Table 1). Lateral transmission of MmuPV1 has been observed in immunodeficient strains of mice experimentally infected with MmuPV1 [15,20]; however, we did not see any warts arise at uninfected sites on the UVB-treated, MmuPV1-infected FVB/NJ mice.

**Table 1:** Papilloma incidence in UVB-irradiated FVB/NJ mice.

Group	UVB Dose mJ/cm^2^	MmuPV1 infection	# Sites infected*	# Sites w/papilloma** (%)
1.	0	Infected	8	0 (0)
		Mock-infected	6	0 (0)
2.	150	Infected	16	0 (0)
		Mock-infected	6	0 (0)
3.	300	Infected	24	14 (58.3)
		Mock-infected	8	0 (0)

* Each ear site infected with 10^8^ VGE.

** Sites scored at the end of 3 months

The presence and size of warts arising on the MmuPV1-infected ears sites of UVB-treated FVB/NJ mice were monitored for a total of 6 months. In this time frame 17% of the sites developed papillomas that completely regressed (Fig 1D and 1E: red lines in Fig 1E). 25% of sites developed papillomas that partially regressed (Fig 1D and 1E-blue lines). Another 17% of sites developed papillomas that continued to grow over the 6 month period (Fig 1D and 1E—green lines). 41% of the sites did not develop papillomas.

To address whether UVB is exerting its effect directly on the virus, we tested whether UVB treatment at 300mJ/cm^2^ 24 hours before infection (h.b.i.) had the same impact on papilloma incidence. Over the initial 4-month period post-infection, papillomas arose with similar frequency as in groups of mice exposed to UVB 24 hours after infection (Fig 1B, *P* = 0.814, log-rank, *two-sided*). Again, we saw complete regression of a similar fraction of papillomas when mice were observed up to 6 months post-infection (Fig 1C). We also found that a subset of animals infected with MmuPV1 and treated with a UVB fourteen days post-infection also developed papillomas at a similar frequency (S1 Table).

To learn if the UVA spectra (315-400nm range UVR) also caused immunocompetent mice to become susceptible to MmuPV1-induced papillomatosis, we infected FVB/NJ mice with 10^8^ VGE of MmuPV1 at sites on the ears and exposed them to 300 J/cm^2^ of UVA 24 hours post-infection. We failed to observe papillomas arise at any sites on these mice (S2 Table). Combining UVA (300 J/cm^2^) and UVB (300 mJ/cm^2^) treatment gave a similar incidence of papillomas as seen with UVB alone (S2 Table).

We also tested the susceptibility of other commonly used, wild-type, inbred strains of mice to MmuPV1-induced disease with varying doses of UVB (S3 Table): 30% of infected ear sites on C57/BL6 mice developed papillomas at the three-month time point when treated with UVB at a higher dose of 600mJ/cm^2^. Only one infected site on a BALB/c mouse developed a papilloma when exposed to 1200mJ/cm^2^ UVB (S3 Table). These results indicate that there are strain differences in susceptibility of mice to MmuPV1-induced disease following UVB irradiation. Because UVB-treated FVB/NJ mice were most susceptible to MmuPV1 disease (p = 0.041 FVB/NJ vs C57/Bl6 or 0.0044 FVB/NJ vs BalbC, at 300 mJ/cm2), we pursued all further studies of the role of UVB in inducing MmuPV1 infection using the FVB/NJ strain of mice.

**Fig 2:**
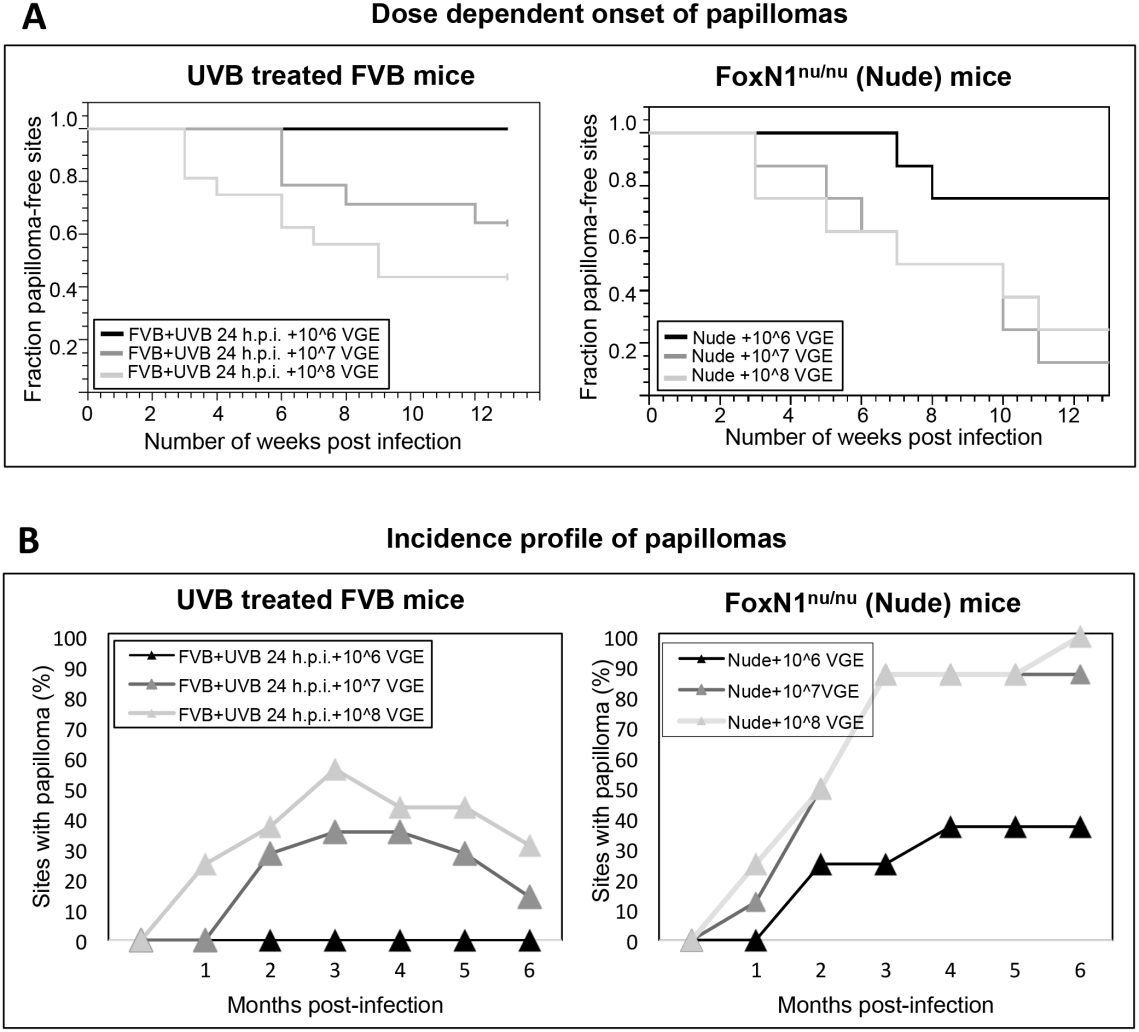
Viral dose-dependent incidence of papillomas in UVB-treated FVB/NJ mice and in FoxN1^nu/nu^ mice. Immunocompetent FVB/NJ mice were infected at ear sites with 10^8^, 10^7^ or 10^6^ VGE of MmuPV1 virions following scarification. Twenty-four hours post-infection (h.p.i.), mice were irradiated with 300mJ/cm^2^ UVB and scored weekly for presence of ear papillomas up to 6 months. In parallel, T-cell deficient FoxN1^nu/nu^ mice not treated with UVB were infected at ear sites with the same stock of virus at designated doses. Graphs for each strain are shown separately. **(A)** Kaplan-Meyer plot of the fraction of papilloma-free infected sites over the initial four-month period following infection. There was no significant difference between incidences of disease between UVB-irradiated FVB mice treated with 10^7^ versus 10^8^ VGE of MmuPV1 (*P* = 0.21, Wilcoxon log-rank test, *two-sided*). FVB/NJ mice infected with 10^6^ VGE of MmuPV1 did not develop any papillomas. **(B)** Percentage of sites with overt papillomas over a 6-month observation period. Note that in both groups, UVB-irradiated FVB mice infected with 10^7^ and 10^8^ VGE of MmuPV1, a subset of papillomas completely regressed by 6 months post-infection (left). There was no regression of papillomas in FoxN1^nu/nu^ mice (right).

To investigate the relationship between viral dose and papilloma incidence in FVB/NJ mice treated with UVB, we infected mice on their ears with 10^8^, 10^7^ or 10^6^ VGE of MmuPV1 and exposed the mice to 300mJ/cm^2^ of UVB 24 hours post-infection (Fig 2). In parallel, T-cell deficient FoxN1^nu/nu^ mice were also infected at ear sites with the same stock of virus at designated doses in the absence of UVB-treatment (Fig 2). By 3 months post-infection, 90% of ear sites in FoxN1^nu/nu^ mice infected with 10^8^ and 10^7^ VGE MmuPV1 developed papillomas (Fig 2A and 2B –Right). At this time-point, papillomas developed in UVB-irradiated FVB/NJ mice infected with 10^8^ and 10^7^ VGE MmuPV1, but at lower penetrance (Fig 2A and 2B—Left) and no papillomas formed on UVB-treated FVB/NJ mice infected with 10^6^ VGE of MmuPV1 up to 6 months post-infection (Fig 2B- Right). In contrast, 37.5% of FoxN1^nu/nu^ mice ear sites infected with 10^6^ VGE of MmuPV1 develop papillomas (Fig 2B-Left). Non-irradiated FVB/NJ mice infected with 10^8^, 10^7^ or 10^6^ VGE of MmuPV1 did not develop any papillomas. These results indicate that a higher threshold in the amount of virus is required to see papilloma induction in immunocompetent animals after UVB irradiation. Furthermore, there was complete regression of a subset of papillomas in FVB/NJ mice infected with 10^7^ and 10^8^ VGE of MmuPV1 when monitored up to 6 months post-infection (Fig 2B). There was no regression of papillomas in immunodeficient FoxN1^nu/nu^ mice.

### MmuPV1-induced papillomas in UVB-irradiated immunocompetent mice show productive infection and signs of neoplastic transformation

**Fig 3:**
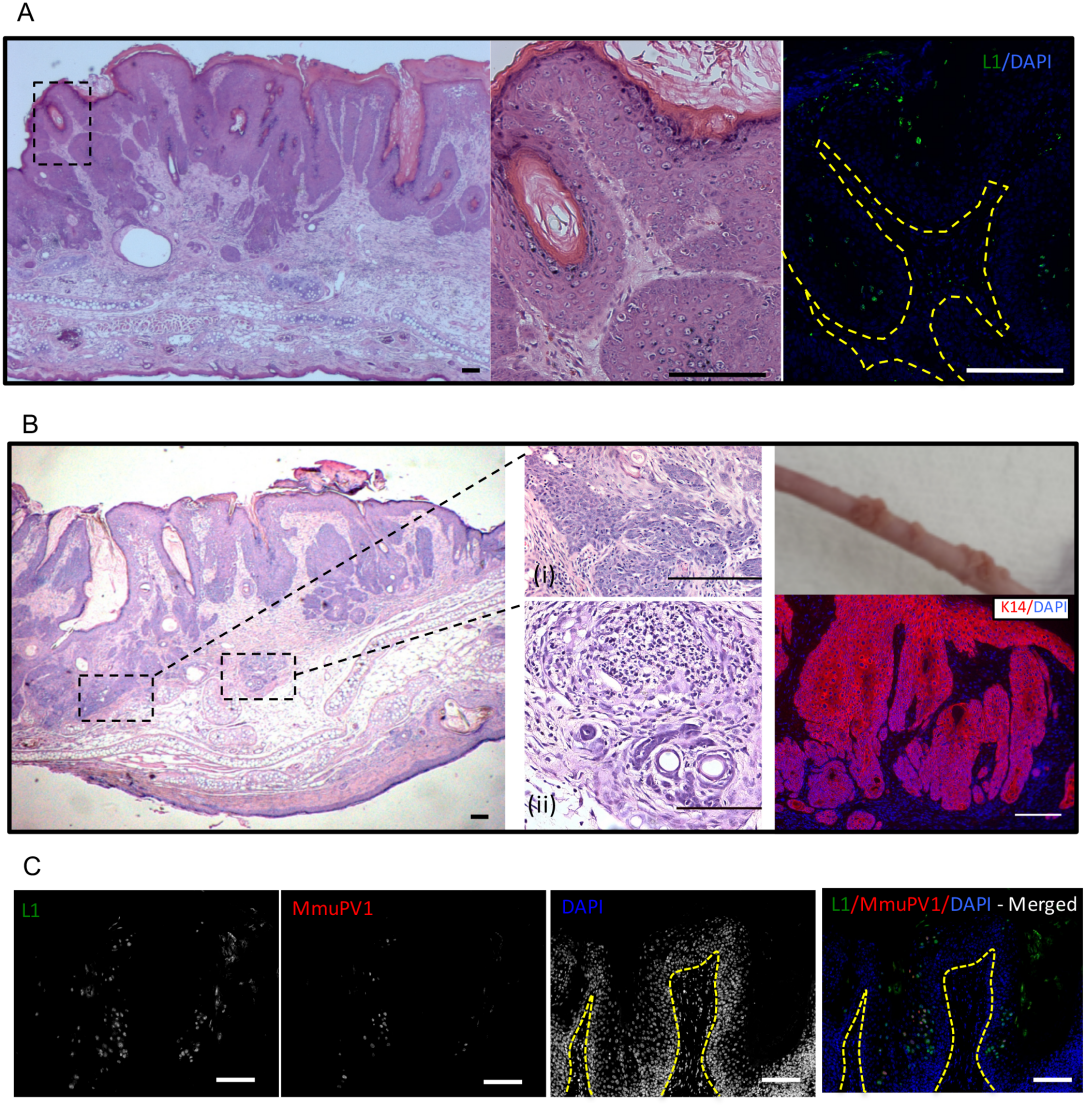
Histopathological analysis of MmuPV1-induced papillomas in UVB –irradiated mice. **(A)** H&E staining of ear tissue indicating presence of a sessile ear papilloma caused by MmuPV1 in FVB mice following UVB irradiation (left). Presence of koilocytes is indicated (middle). L1-immunofluorescent staining of papillomas (right). **(B)** Sessile papilloma with areas indicating frank malignant transformation (inset;i) and foci of inflammation (inset;ii). K14 staining of papilloma and associated epithelial invasion into underlying dermis. Papillomas on the tail of an immunodeficient BALB/c *FoxN1* ^*nu*^ mice (right, top). These papillomas were formed as a result of infection with viral extracts prepared from papillomas that arose in immunocompetent FVB mice infected with MmuPV1 followed by UVB irradiation. **(C)** L1 (green)-MmuPV1 FISH (red) co-staining of MmuPV1-induced ear papillomas in UVB irradiated FVB/NJ mice. The nuclei were counterstained with DAPI (blue). Scale bars represent 100μm.

Lesions arising on the ear sites of MmuPV1-infected FVB mice treated with UVB were harvested at 6 months post-infection and analyzed histopathologically. They showed sessile papilloma-like morphology with hyperkeratosis (Fig 3A, left; S1 Fig-Panel B). Cells within the stratum granulosum showed presence of koilocytes consistent with productive papillomavirus infection (Fig 3A, middle). The MmuPV1 major viral capsid protein was expressed in these papillomas as indicated by L1-specific immunofluorescence (Fig 3A, right). Viral capsid protein was mostly detected in the koilocytes and other cells within the upper layers of the epithelia (suprabasal and terminally differentiated) with some L1-positive cells occasionally observed in the basal layer consistent with prior findings in immunodeficient mice [19]. The sessile papillomas were accompanied by multiple areas of atypical squamous cell hyperplasia, several of which were suggestive of early neoplastic transformation, consistent with other reports [17,19,20]. In our case, however, we also found focal areas of malignant progression consistent with squamous cell carcinoma with invasion extending into follicular structures deep within the dermis as evident from cytokeratin-14 staining (Fig 3B). These areas of malignant progression were observed in several histopathologically scored lesions (e.g. see S1 Fig- Panel B). Foci of chronic inflammation were also noted in the lesions, some of which extended into the atypical dermis (Fig 3B).

Southern blot quantification showed that viral extracts harvested from papillomas from immunocompetent mice had a 1000-fold reduction in the amount of VGE/mg of wart harvested when compared to the papillomas arising in immunocompromised *FoxN1* ^*nu*^ mice (S2 Fig). Regardless, the viral extracts from immunocompetent mice were infectious and caused papillomas in *FoxN1* ^*nu*^ mice (Fig 3B). MmuPV1 DNA-specific *in situ* hybridization coupled with L1 immunohistochemistry analysis of MmuPV1-induced ear papillomas in UVB irradiated FVB/NJ mice (Fig 3C) showed presence of amplified viral DNA and L1 capsid. Robust L1 expression was seen throughout the papilloma most frequently in the suprabasal layers of the epithelia. The FISH positive cells were comparatively less frequent and predominantly seen in the spinous epithelium occasionally showing co-localization with L1 positive nuclei. We observed that while regions of papillomatosis showed presence of amplified MmuPV1 DNA areas of malignancy showed little to no presence of MmuPV1 amplified DNA (S3 Fig). Presence of the viral major capsid protein (L1) and amplified viral DNA, which are markers for the productive stage of the viral life cycle [reviewed in [29]], coupled with findings from the transmission experiments (Fig 3B), confirm that MmuPV1 establishes a productive infection in immunocompetent mice following UVB treatment.

### UVB irradiation has a systemic effect on host biology

**Fig 4:**
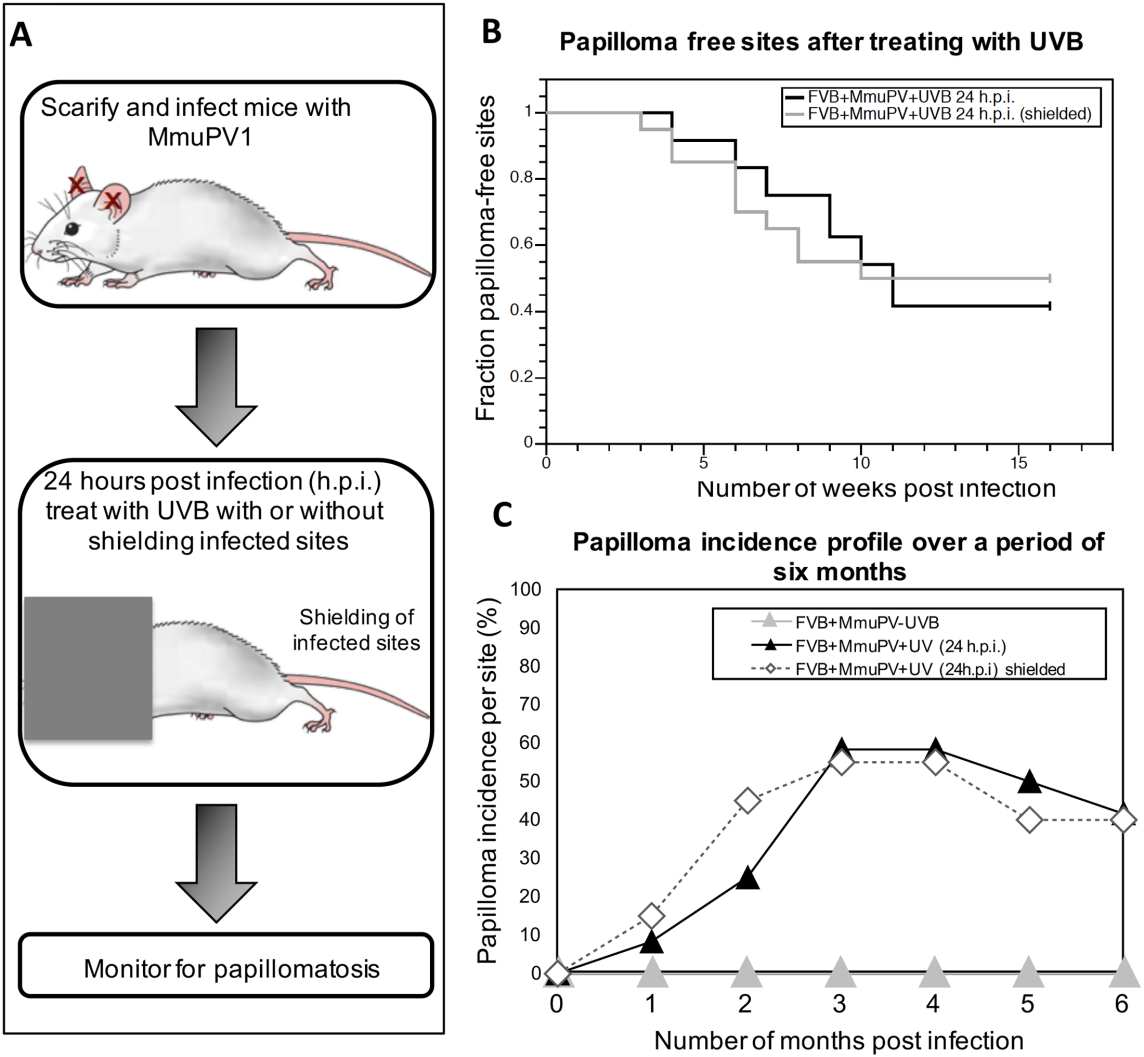
Systemic effect of UVB assists in MmuPV-dependent papillomatosis. **(A)** Schematic illustrating the experimental design. Twenty-four hours post infection with 10^8^ VGE, groups of mice were either whole body UVB irradiated (FVB+MmuPV+UVB 24h.p.i.) or infected sites were shielded from UVB (FVB+MmuPV+UVB 24h.p.i. shielded) and scored weekly for presence of ear papillomas up to 6 months. UVB dose was 300mJ/cm^2^. Scoring data is compared to the FVB+MmuPV+UVB 24h.p.i. group also shown in Fig 2, as both experiments were performed at the same time. **(B)** Kaplan-Meyer plots of the fraction of papilloma-free infected sites over the first 12 weeks post-infection. There was no significant difference between the two experimental groups (shielded versus unshielded ears) (*P* = 0.938) as assessed by Wilcoxon log-rank analysis. **(C)** Percentage of sites with overt papillomas over a 6-month observation period. Some papillomas completely regressed in both groups. Control animals i.e. non-UVB irradiated FVB mice infected with MmuPV1 (FVB+MmuPV-UVB) did not develop any papillomas over the 6 month observation period.

There are several lines of evidence in the field of photoimmunology indicating that UVB impairs a variety of immune responses in humans and laboratory animals both locally, within UV-irradiated skin, and systemically, at distant sites [30–32]. To test whether UVB-assisted pathogenesis in MmuPV1-infected FVB/NJ mice is due to a systemic or a local effect of UVB-irradiation, we infected FVB/NJ mice at ear sites as described previously. Twenty-four hours after infection, mice were anesthetized and the infected ear sites were shielded with tin foil leaving the rest of the mouse exposed to 300mJ/cm^2^ UVB irradiation (Fig 4A). Mice were then observed weekly to score for papillomatosis up to six months post-infection. At the end of 16 weeks approximately 55% of infected mice developed papillomas on ear sites (Fig 4B). There was no significant difference in the temporal onset of papillomas between shielded and unshielded mice (*P* = 0.938, log-rank, *two sided*). The animals were kept under observation for 6 months and scored weekly for papilloma incidence. We found that by six months post-infection 27% of the papillomas completely regressed in the mouse cohort whose infection sites were shielded, similar to that seen in the unshielded cohort, where 28.5% of the papillomas had completely regressed. These results indicate that systemic effects of UVB on host biology must contribute to the ability of UVB to induce MmuPV1-dependent disease.

### UVB-mediated immunosuppression correlates with development of MmuPV1- dependent cutaneous disease

**Fig 5:**
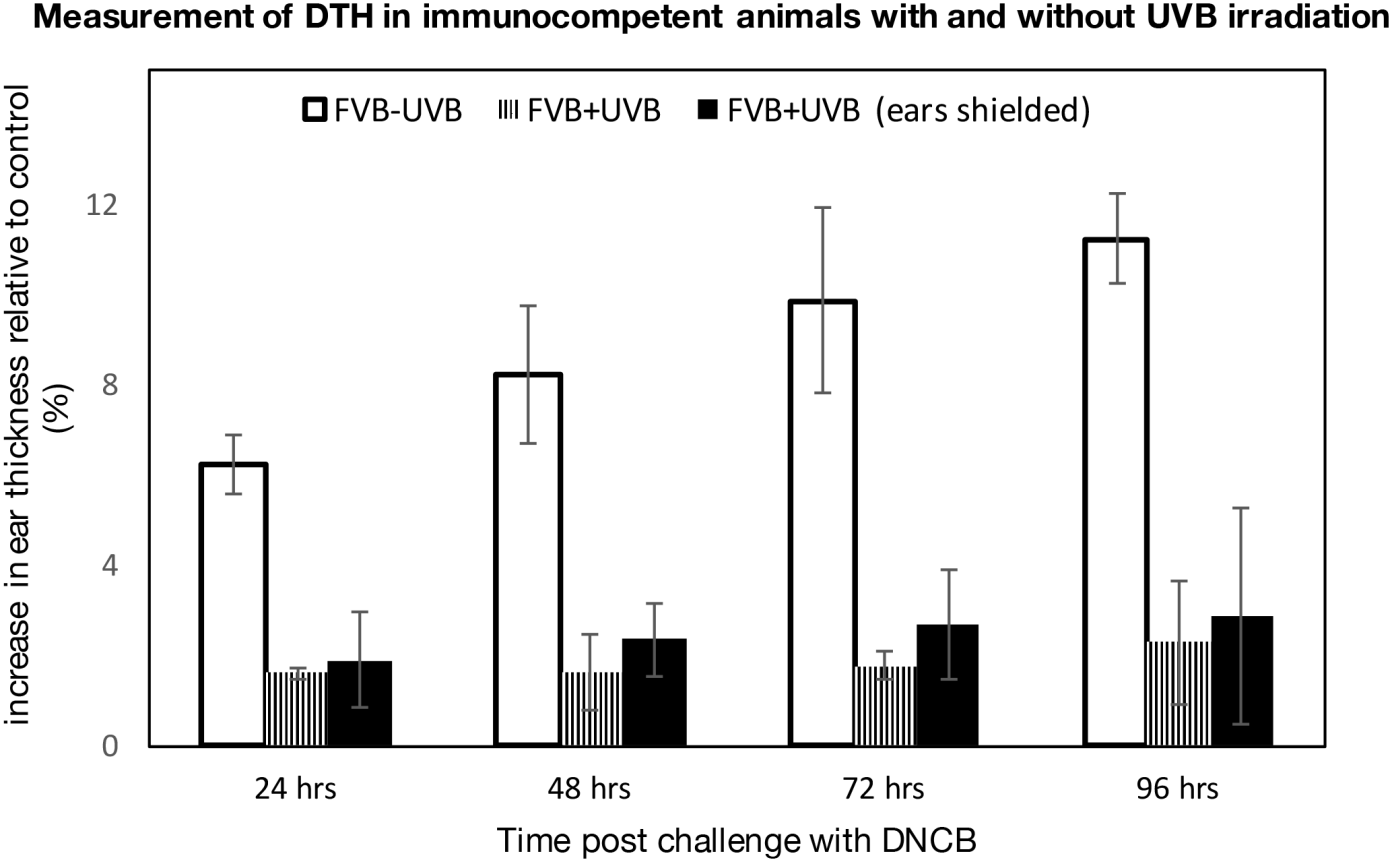
UVB causes systemic immunosuppression of host. Three groups of mice were treated as follows: a control group that was not UVB irradiated (FVB-UVB, White bars, n = 3), UVB-irradiated group (FVB+UVB, Striped bars, n = 6) and UVB-irradiated group in which ears were shielded from UVB exposure [FVB+UVB (ears shielded), Black bars]. Ear thickness reported as the average of the difference between DNCB-challenged (left) and unchallenged (right) ears four days post challenge. SEM refers to standard error in measurement computed by measuring standard deviation. DTH measured by the change in thickness of the ear (ΔEar) was found to be statistically significant between UVB-treated mice and non-irradiated control mice (student t-test, *p<0*.*005*, *two-sided)* for both UVB-treated groups.

Through the study of different strains of immunodeficient mice, it has been found that T-cell deficiency is necessary for the development of MmuPV1-dependent papillomas [18,20]. Several studies have demonstrated that UVB causes cell-mediated immunosuppression in mice [33,34], reviewed in [30,32,35,36]. Cell-mediated immunosuppression by UVB has traditionally been measured by monitoring delayed type hypersensitivity (DTH) responses [30,32,35,37,38]. A single exposure to UVB irradiation is sufficient to inhibit DTH responses in some strains of mice [39,40]. To determine if the single dose of UVB irradiation (300mJ/cm^2^) that makes FVB/NJ mice susceptible to MmuPV1-induced papillomatosis (Table 1) is sufficient to cause immunosuppression in this strain of mice, we measured DTH responses in these and control mice not treated with UVB [41,42]. Ten days post-UVB treatment, FVB/NJ mice were sensitized to an antigen by topically applying 0.5 mg 1-Chloro-2, 4-Di-Nitrobenzene (DNCB) on the shaved backs of the mice. Five days later we determined the level of immune response to this antigen by applying 0.2 mg DNCB to a distant site (the ears) and monitoring DTH responses (Fig 5). DTH was assessed by measuring ear swelling every 24 hours for four days. A single exposure to UVB (300mJ/cm^2^) was capable of causing immunosuppression in FVB/NJ mice as evidenced by a marked decrease in swelling in response to DNCB challenge in the UVB treated mice compared to non-UVB treated mice (Fig 5). This difference was statistically significant (*p<0*.*005*, T-test). To assess whether this immunosuppression was systemic or local, we repeated the DTH assay on mice in which we shielded their ears from UVB as described previously. These mice also displayed reduced swelling in response to challenge with DNCB on their ears, indicating that UVB-induced immunosuppression in these mice is systemic (Fig 5). This observation suggests that UVB causes systemic immunosuppression that may assist MmuPV1-dependent disease (Figs 1–4).

### Mice displaying long-term UVB-induced immune suppression preferentially develop MmuPV1-induced warts

**Fig 6:**
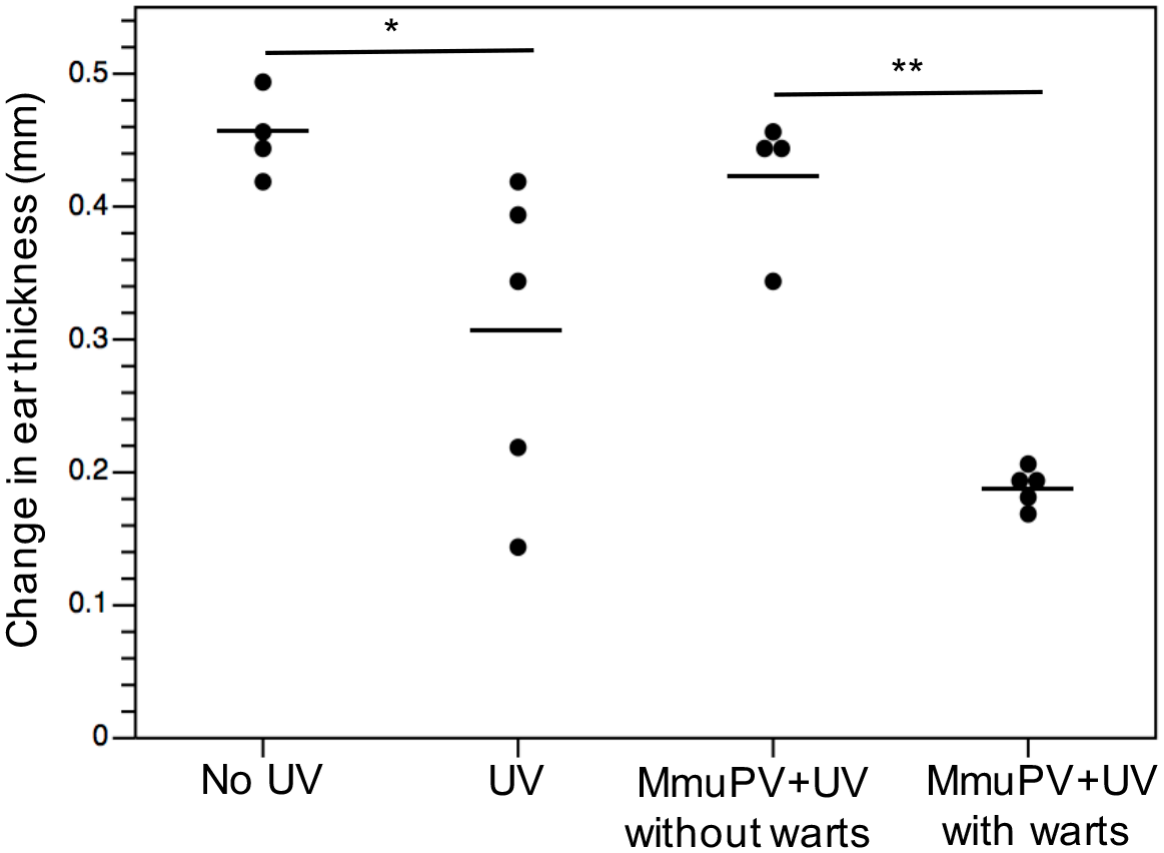
Long-lived immune suppression in UVB-irradiated mice correlates with papilloma incidence. Mice were either infected with MmuPV1 or vehicle followed by UVB irradiation (300mJ/cm^2^) in the right ear. Mice were sensitized with DNCB 10 days post UVB exposure and were challenged DNCB in the left ear 3 months following infection. Ear swelling was measured by means of a Vernier calipers. Ear thickness is reported as the average of the difference between ear thickness 0 hrs post challenge and 72 hrs post challenge. Ear thickness for each mouse is shown as a dot plot. The black lines represent the mean reading for each group. Wilcoxon rank-sum test was used to analyze difference between several groups. There was significant difference between UVB-irradiated (UV) and control (no UV) groups (*p = 0.021, *two-sided*). There was significant difference between the UVB irradiated animals infected with MmuPV1 that developed warts and those that did not develop warts (**p = 0.016, *two-sided*). There was no significant difference between control (no UV) and UVB-irradiated mice infected with MmuPV1 that did not develop warts (MmuPV1+ UV without warts).

To determine if UVB-mediated immune suppression correlates with papilloma incidence we performed long-term DTH assays in FVB/NJ mice following infection with MmuPV1 and UVB-treatment. Mice were infected with MmuPV1 in their right ear and exposed to a single dose of UVB (300 mJ/cm^2^) 24 hrs post-infection as described previously (Fig 1A). Ten days post UVB-treatment animals were sensitized to antigen by painting 0.5 mg of DNCB on their shaved backs. Mice were monitored for papilloma formation. Three months post infection, animals were challenged with 0.2 mg DNCB on the uninfected, left ear. We chose the three-month time period because this is the time by which FVB/NJ mice treated with UVB develop the maximal number of MmuPV1-induced warts (Figs 1C and 2B). Post-challenge we measured ear swelling up to 96 hrs (Fig 6). We found that in the control group (animals not treated with UVB) a long-lived DTH response was established as evidenced by swelling of the ears. UVB-treated, uninfected control mice displayed a range in levels of DTH response, indicative of variable levels of long-term immune suppression. This was also seen in UVB-treated, MmuPV1-infected mice. Of specific note, the mice in this latter group that retained immune-suppression at the end of 3 months were the same ones that had developed MmuPV1-induced warts. The difference in ear swelling between the UVB-irradiated animals infected with MmuPV1 that developed warts and those that did not develop warts was statistically significant (*P* = 0.016, Wilcoxon rank-sum, *two-sided*). These data demonstrate a strong correlation between long-term, UVB-induced immunosuppression and MmuPV1-dependent pathogenesis.

## Discussion

The lack of infection models in a tractable laboratory animal has limited our ability to study the pathogenesis of papillomaviruses in their natural hosts. The murine papillomavirus, MmuPV1, isolated from cutaneous warts arising on immunodeficient *NCR-FoxN1* ^*nu/nu*^ laboratory mice [15,16] is a valuable animal papillomavirus because it provides us, for the first time, the opportunity to study papillomavirus infections in the context of a genetically manipulatable host.

Prior to this study, MmuPV1-associated papillomatosis has been described primarily in the context of immunodeficient strains of mice [17,18,20,21], though there have been reports that MmuPV1 can cause warts on hairless strains of mice, which are thought to be immunocompetent [15,43]. Consistent with a role of the host immune system in limiting MmuPV1-induced disease, Handisurya, *et al*. (2014) found that Cyclosporin A treatment is required for induction and maintenance of MmuPV1-induced papillomas in immunocompetent mice [18]. These authors did find that, at very high doses of virus (10^12^ VGEs), the SENCAR strain of mice, selectively bred for high susceptibility to skin tumor induction by chemical carcinogens [44], developed papillomas, but these papillomas regressed within two weeks of appearing.

In this study we investigated whether the UVB spectra impacts susceptibility of immunocompetent mice to MmuPV1. There was limited prior evidence suggesting the role of UV radiation in other animal models for papillomavirus infection. *Mastomys natalensis* Papillomavirus (MnPV) is a rodent papillomavirus that is shown to cause papillomatosis in the African multimammate rat [25]. MnPV DNA was found in UV induced tumors in HRA/Skh mice that are hairless but immunocompetent [26]. Further, studies showed that cell-free extracts containing MnPV enhanced UV-induced tumorigenesis [45]. We found that when FVB/NJ mice were exposed to high doses of UVB (Table 1) greater than 50% of infected sites developed papillomas by 3 months, some of which persisted for 6 months (Figs 1–4). UVB also induced MmuPV1-dependent papillomatosis in other strains of immunocompetent mice (S3 Table).

Histopathological analysis of the papillomas in the MmuPV1/UVB infection model also indicated progression to squamous cell carcinoma (Fig 3, S1 Fig-Panel B). Interestingly, we observed that while regions of papillomatosis showed many cells that had amplified viral DNA, we only observed a few cells harboring amplified viral DNA in papilloma-associated malignant regions (S3 Fig). More sensitive *in situ* hybridization techniques will be required to determine whether MmuPV1 viral genomes are lost as malignant progression arises, as is thought to occur in beta-HPV associated non-melanoma skin cancers [46].

Several lines of evidence supports the hypothesis that UVB is having an indirect effect on increasing the susceptibility to MmuPV-1 induced papillomatosis by inducing systemic immunosuppression. First, we did not find any significant difference in susceptibility to wart formation in mice that were treated with UVB 24 hours before or 24 hours after infection with MmuPV1 (Fig 1). Second, UVB's ability to increase susceptibility to MmuPV1-induced papillomatosis was equally efficient whether or not the site of infection was exposed to UVB (Fig 4). And third, long-lived immunosuppression correlated with wart formation (Fig 6).

A second but not mutually exclusive hypothesis is that UVB also directly influences MmuPV1-induced pathogenesis. Supportive of this hypothesis, expression of the viral genes in K14HPV8 transgenic mice were increased following exposure to UVR [47]. The physiological relevance of this observation remains unclear, however, because the HPV8 genes are under the control of a heterologous, keratin 14 promoter in the context of this transgenic mouse model. The MmuPV1 infection model should provide a valuable experimental platform for further testing this second hypothesis.

The ability of papillomaviruses to persist in their host in the absence of causing overt disease, i.e. latency, has long been suspected. Compelling evidence for latency comes from the observation that cyclosporine-induced immunosuppression led to elevation of the viral DNA copy number at sites of wart regression in cottontail rabbits infected with rabbit oral papillomavirus, consistent with reactivation of virus from latency [48]. In the MmuPV1-infection model, ELISA analysis of serum from C57BL/6 mice infected with MmuPV1 showed seroreactivity to MmuPV1 virus particles at 70 days post-infection even though they did not develop warts [49]. Likewise, seroconversion was seen in all mice (n = 20) in a group of SKH-1 mice infected with MmuPV1, of which 3 mice actually developed papillomas [43]. Together, these observations indicate that the virus is presented to the immune system even in the absence of causing overt disease. Whether this results from the original exposure of the animals to the virus or the consequence of a latent infection remains unclear. Our own observation that a single exposure to UVB 14 days post-infection led to papillomatosis at sites infected with MmuPV1 (S1 Table) indicates either that infectious virus is stably retained at the site of infection for that period of time and then initiates infection post UVB exposure or that latent infections arose that were then activated by UVB-induced immunosuppression. It remains to be determined whether latency arises in the MmuPV1 infection model, and, if so, the nature of this latency.

In this study we observed an unexpected tissue specificity in terms of susceptibility to MmuPV1-induced papillomatosis; while infection of the ear led to efficient formation of papillomas in UVB-treated FVB/NJ mice, papillomas did not arise at tail sites that were infected in the same mice. Others have reported that there is some site specificity for MmuPV1-induced disease in immunodeficient mice [19,20]. For example, while tail and muzzle of immunodeficient mice are susceptible to MmuPV1-induced papillomatosis, the torso skin is not. There are limited studies that have been directed towards looking at difference in susceptibility of different cutaneous sites of mice to cancer. Therefore the molecular differences between the sites are not very clear. In our studies with HPV 16 transgenic (K14E6, K14E7) mice we have consistently observed epithelial hyperplasia most extensively in the ear skin [50–52]. Likewise, ear skin of HPV 38 E6/E7 transgenic mice display patches of hyperproliferation [53]. Recently, there has been one report that suggests that difference in miR-155 expression of ear versus chest/torso skin of K14HPV16 mice could explain the difference in susceptibility of different tissues to develop HPV-mediated carcinogenesis [54]. It is also possible that grooming behaviors of mice, such as scratching of ear, promotes wounding [55] thereby allowing virus to access lower layers of epithelia better.

In our studies we observed a clear viral-dose dependence in causing disease. Interestingly, we found that a viral dose of 10^7^ VGE or greater was required to see papillomas in UVB-irradiated FVB/NJ mice whereas 10^6^ VGE of MmuPV1 were sufficient to induce papillomas on BALB/c-*Foxn1* ^*nu/nu*^ mice, albeit at low incidence over the same 6-month observation period (Fig 2). This suggests that there is a higher threshold of virus required to see MmuPV1-dependent disease in UVB treated immunocompetent animals. We posit that this observation reflects, at least in part, the fact that long-term immunosuppression induced by UVB in FVB/NJ mice only occurs in a subset of the UVB-treated mice (Fig 6).

In our testing of different genetic backgrounds, only FVB/NJ was susceptible to MmuPV1-associated pathogenesis at 300mJ/cm^2^, whereas C57/BL6 and BALB/c mice were susceptible to MmuPV1-associated pathogenesis at higher doses of UVB (S3 Table). This observation is consistent with a previous study that suggests that different genetic backgrounds respond differently to UVB in terms of levels of immunosuppression based upon DTH assays [56]. While that study did not test FVB/NJ mice, they found that C57/BL6 mice were more sensitive to UVB-induced immunosuppression than BALB/c mice. This correlates with the level of MmuPV1-dependent papillomatosis observed in our studies (S3 Table).

In our study we observed that UVB, but not UVA alone makes immunocompetent mice susceptible to MmuPV1-dependent papillomatosis (S2 Table). UVB has been shown to play a key role in initiating and mediating immunosuppression, whereas the mechanisms and roles of UVA in immunosuppression are not well understood [57,58]. UVB can cause both short-term as well as long-term defects in cell-mediated DTH responses [35,38,40], at least in part by inhibiting development of memory T-cells and by causing an overall reduction in T-cell subpopulations in the skin [59]. We observed that UVB caused variable levels of long-term immunosuppression in FVB/NJ animals and that MmuPV1-induced papillomas developed preferentially in those animals retaining long-term immunosuppression (Fig 6). This can explain the observation that papillomas arise in approximately 50% of animals. Based upon these findings we also posit that the observed regression of papillomas (Figs 1–2) correlates with a loss of immunosuppression.

The observation that there is a strong correlation between long-term immunosuppression induced by UVB and MmuPV1-dependent pathogenesis is perhaps the most notable finding of this study. This correlation supports the hypothesis that UVB-induced immunosuppression can help drive papillomavirus-induced disease. There is correlative epidemiological data from human studies in which anatomical sites on individuals that are exposed to sunlight, or at which sunburn has occurred are increased in their susceptibility to HPV-induced warts. One difference however, is that in our studies with FVB/NJ mice, the effect of UVB was found to be systemic; UVB irradiation did not have to be applied to the infection site. This raises the interesting question: is there a difference in the role of UVB in HPV-driven pathogenesis compared to MmupV1-driven pathogenesis? Further studies are needed to assess whether a local effect of UVB on MmuPV1 can be identified in mice. Recently, it has been shown that MmuPV1 also infects the mucosal epithelium of the female reproductive tract and oral cavity [21]. In this regards MmuPV1 infection model is truly unique and the systemic immunosuppression by UVB can further be tested in the context of mucosal disease.

In conclusion, we have reported the novel finding that UVR makes immunocompetent mice susceptible to development of MmuPV1-induced cutaneous papillomas, and this correlates with UVB-induced systemic immunosuppression. This observation opens the door to pursuing studies using genetically engineered mice to study molecular pathways that mediate the role of UVB in making mice susceptible to papillomavirus-induced pathogenesis, as well as identifying cellular targets of MmuPV-1 encoded factors that mediate their role in pathogenesis.

## Materials and Methods

### Animals

The following mice were obtained and bred for the purpose of this study (vendor in parenthesis): immunocompetent FVB/NJ (Taconic), BALB/c (Charles River), C57BL/6 (Jacksons Lab); immunodeficient athymic BALB/c FoxN1nu/nu (Harlan). All infected mice were housed in aseptic conditions in micro-isolator cages. Animals were handled only by designated personnel and personal protection gear was changed between cages to prevent any cross contamination from virus.

### Ethics statement

Mice were housed at McArdle Laboratory Animal Care Unit in strict accordance with guidelines approved by the Association for Assessment of Laboratory Animal Care, at the University of Wisconsin Medical School. All protocols for animal work were approved by the University of Wisconsin Medical School Institutional Animal Care and Use Committee (Protocol number: M02478).

### MmuPV1 virus stock

MmuPV1 virus stock was generated by isolating MmuPV1 virions from papillomas in nude mice as described previously [18,19,60]. The MmuPV1 infection model was established in nude mice using quasivirions generated (as described previously [19,61]) using a clone of MmuPV1 obtained in the Lambert lab. Briefly, this clone of MmuPV1 was made by performing rolling circle amplification on the virus extracts (generously provided by Dr. Aravind Ingle, ACTREC, India) from the original colony of nude mice infected with MmuPV1 [15] followed by cloning into the pUC19 vector [15,16]. To encapsidate MmuPV1 genome we used pMusSHELL, a Mammalian expression plasmid with codon modified L1 and L2 genes of MmuPV1 (generously provided by Dr. Chris Buck, NIH) [60]. To confirm that the virus stock was infectious, nude mice were infected in parallel during each experiment as positive controls.

### Isolation of infectious MmuPV1 virions

This protocol has been modified from previously described methods of isolating non-enveloped viruses from human skin or tissue samples [62,63]. Animals with warts were euthanized and 10 mg of excised wart was homogenized in 700 μl PBS containing Triton-X-100 (1%). Benzonase was added to the homogenized wart sample and incubated at 37°C for 30 minutes. Collagenase H (2 mg) was added, and the sample was vortexed and then incubated at 4°C overnight. Sodium chloride concentration was adjusted to 0.8M, the sample was centrifuged for 5 minutes at 5000g, and the supernatant was clarified by ultracentrifugation through an Optiprep (iodixanol) step gradient followed by fractionation as described in detail on the following website: http://home.ccr.cancer.gov/LCO/pseudovirusproduction.htm. Viral genome equivalence was estimated by comparing the amount of encapsidated viral DNA in the viral stock, liberated by treatment with proteinase K, to known standards of cloned MmuPV-1 genome by Southern analysis using MmuPV1-specific probes, followed by quantification using ImageJ software (S1 Text).

### MmuPV1 infection model

*In vivo* infection with purified MmuPV1 virions was performed on scarified skin of the animals' ears. Animals were anesthetized and tails or inner ears were scarified using a 27-gauge syringe needle to scrape the epithelia (not sufficient to cause bleeding) followed by pipette delivery of virus solution using a siliconized pipette tip. This method is modified from a previously reported infection model [60]. As controls mice were mock infected with vehicle i.e. optiprep as described above.

### UV treatment

Animals were exposed to a single dose of UVB using a custom designed Research Irradiation Unit (Daavlin, Bryan, OH) [64–66]. This irradiation unit consists of an exposure unit mounted on fixed legs. Within the exposure unit there are four UVA and UVB lamps controlled using Daavlin Flex Control Integrating Dosimeters. In this system, dose units can be entered in milli-Joules per Centimeter Square for UVB (mJ/cm2) and Joules per Centimeter Square for UVA (J/cm2); variations in energy output are automatically compensated to deliver the desired dose. This enables us to expose the animals to an accurate dosimetery of UVB radiation. For accuracy, the machine is periodically calibrated using International Light IL 1400, digital light meter (Daavlin Company). For ear shielding experiments, ear sites were shielded from UV exposure by covering the head of anesthetized mice with tin foil during UV exposure.

### Papilloma scoring and size determination

Papillomas were measured bi-weekly. Fraction of papillomas that completely regressed was computed by expressing number of papillomas at end-point of study (i.e. 6-months post infection) compared to maximum number of papillomas formed (i.e. papillomas at 3-months post infection). Papilloma size was determined by calculating the cubic root of the product of length × width × height to obtain a geometric mean diameter (GMD) as described previously [67]. Data were represented as the means (± standard errors of the mean [SEM] of the GMDs for each test group.

### Delayed Type Hypersensitivity assays

DTH assays were performed by topically applying 1-Chloro-2,4-Di-Nitrobenzene (DNCB) (Sigma) using a modification of a method described previously [41]. For short-term immunosuppression studies, mice irradiated with 300mJ/cm^2^ UVB. Ten days post UVB-irradiation mice were shaved on their backs and sensitized by topically applying 0.5% DNCB in 50μl vehicle (4:1 acetone:olive oil). Five days post-sensitization they were challenged with DNCB in the left ear and ear thickness was measured. To assess correlation between wart incidence and long-term immunosuppression mice were either infected with MmuPV1 or mock infected in the right ear followed by treatment with 300mJ/cm^2^ UVB. Mice were sensitized with DNCB (0.3% in 50μl vehicle) 10 days post UVB exposure on their backs. Three months post-sensitization, mice were challenged with DNCB in the left ear and ear thickness was measured. Ear thickness was measured by means of Vernier calipers up to 96 hrs. The average of three readings taken at different points across the ear swelling was considered as a single measurement. Ear thickness was reported as the average of the difference between challenged and control ears 24–96 hours post challenge. The control (right) ear was the ear that was challenged with the vehicle only. The standard error in measurement was computed by measuring standard deviation.

### Histology

Skin was harvested, fixed in 4% paraformaldehyde, and embedded in paraffin. Serial sections (5 μm thick) were stained with hematoxylin and eosin (H&E) and evaluated for histopathological features. Immunofluorescent staining was performed on sections after deparaffinizing with xylenes and rehydrating with graded ethanol, respectively. For cytokeratin14 staining, sections were blocked for 1hour at room temperature with goat serum followed by incubation with K14 antibody (Covance) at 1:1000 dilution for 1hour at room temperature. K14 signals were detected with Alexa-fluor 594 against rabbit. For L1-FISH dual immunofluorescent staining, first L1 immunofluorescent staining was performed. Antigen retrieval was performed using Proteinase K (20 μg/ml) for 15 minutes at 37°C. Samples were blocked for 1hour at room temperature with 5% goat serum and incubated overnight at 4°C with rabbit polyclonal immune serum directed against MusPV1 L1 [18,19] at 1:5000 dilution (Gift from Dr. Chris Buck, NCI). To proceed with MmuPV1 fluorescent *in situ* hybridization (FISH), the L1 stained tissue was dehydrated using a series of ice-cold ethanols (70%, 80%, 95%) for 2 minutes each. Slides were dried by placing them in an empty container at 50°C for 5 minutes and then placed in denaturation solution (28 mL formamide, 4 mL 20X SSC pH 5.3, 8 mL water) at 72°C for 2 minutes. The ethanol series was repeated, sections were dried, and denatured digoxigenin (DIG-11-dUTP, Roche)-labeled probe was hybridized to cells overnight at 37°C in a humidified chamber. To make the probe, nick translation was used to label MmuPV1-plasmid DNA with digoxigenin. After washing with 2X SSC and 50% formamide at 50°C (for 30 minutes twice) and 2X SSC at 50°C (for 30 minutes twice), MmuPV1 FISH DNA signals were detected with a digoxigenin-specific antibody conjugated to fluorescein isothiocyanate (Sigma, F3523) at 2% by volume in STM solution (4X SSC, 5% non-fat dried milk, 0.05% Tween-20, 0.002% sodium azide) for 30 minutes at 37°C. L1 protein signals were detected using Alexa-fluor 488 antibody against rabbit. Nuclei were counterstained with DAPI. All images were captured using a Zeiss AxioImager M2 microscope and AxioVision software version 4.8.2 (Jena, Germany).

### Statistical analysis

All statistical analyses were performed using MSTAT statistical software version 6.1.4 (http://www.mcardle.wisc.edu/mstat).

## Supporting Information

S1 Fig **(A) Papillomas on ears of immunocompetent FVB/NJ mice.** Examples of papillomas that arose on ears of FVB/NJ mice infected with 10^8^ VGE MmuPV1 followed by irradiation with 300mJ/cm^2^ UVB. These images represent papillomas at 6 months post infection. (B) Histopathology of MmuPV1-induced ear papillomas in UVB irradiated FVB/NJ mice. Top panel shows H&E images (taken using a 2.5X objective) of lesions. Bottom panel with insets (taken using a 20X objective) indicate corresponding areas of lesions showing focal regions invasivity within the underlying dermis. Scale bars denote 100μm.
(TIF)

S2 Fig Quantification of Viral Genome Equivalence by Southern Hybridization. Southern analysis used to quantify virus stocks obtained from MmuPV1 induced ear warts in immunodeficient nude mice and UVB-irradiated FVB/NJ mice.
(TIF)

S3 Fig Fluorescent in *situ* hybridization to detect MmuPV1 in papilloma and malignant regions. MmuPV1-FISH was performed on papillomas that arose on ears of FVB/NJ mice infected with 10^8^ VGE MmuPV1 followed by irradiation with 300mJ/cm^2^ UVB. Areas of papillomatosis show presence of amplified viral DNA (red) whereas cancerous regions show little to no presence of amplivied viral DNA. Nuclei were counterstained with DAPI (blue). Scale bars denote 100μm.
(TIF)

S1 Table Papilloma incidence in UVB-irradiated FVB/NJ mice infected with MmuPV1 at different time points. FVB/NJ mice were either treated with UVB twenty-four hours prior to infection (Group 1), twenty-four hours post-infection (Group 2) or fourteen days post-infection (Group 3) with MmuPV1. All animals were infected with 10^8^ VGE MmuPV1. UVB dose was 300mJ/cm^2^. Data shown here represents sites scored at 3 months post-infection.
(TIF)

S2 Table Papilloma incidence in immunocompetent mice infected with MmuPV1 followed by UVA or UVB irradiation. FVB/NJ mice were either treated with UVA alone (Group 1), UVB alone (Group 2), or both UVA and UVB (Group 3) twenty-four hours post-infection with 10^8^ VGE MmuPV1. UVB dose was 300mJ/cm^2^ and UVA dose was 300J/ cm^2^. Data shown here represents sites scored at 3 months post-infection.
(TIF)

S3 Table Papilloma incidence in different strains of immunocompetent mice infected with MmuPV1 followed by UVB irradiation. Mice of different genetic backgrounds were infected with 10^8^ VGE MmuPV1 and irradiated with designated dose of UVB twenty-four hours post-infection. Data shown here represents sites scored at 3 months post-infection.
(TIF)

S1 Text Detailed protocol for Southern analysis. Text accompanying materials and methods.
(PDF)
