# Supplementary figures and images for: Role of Ultraviolet Radiation in Papillomavirus-Induced Disease
Source: PLoS Pathog. 2016 May 31;12(5):e1005664. doi: 10.1371/journal.ppat.1005664 (PMC4887022; doi:10.1371/journal.ppat.1005664)

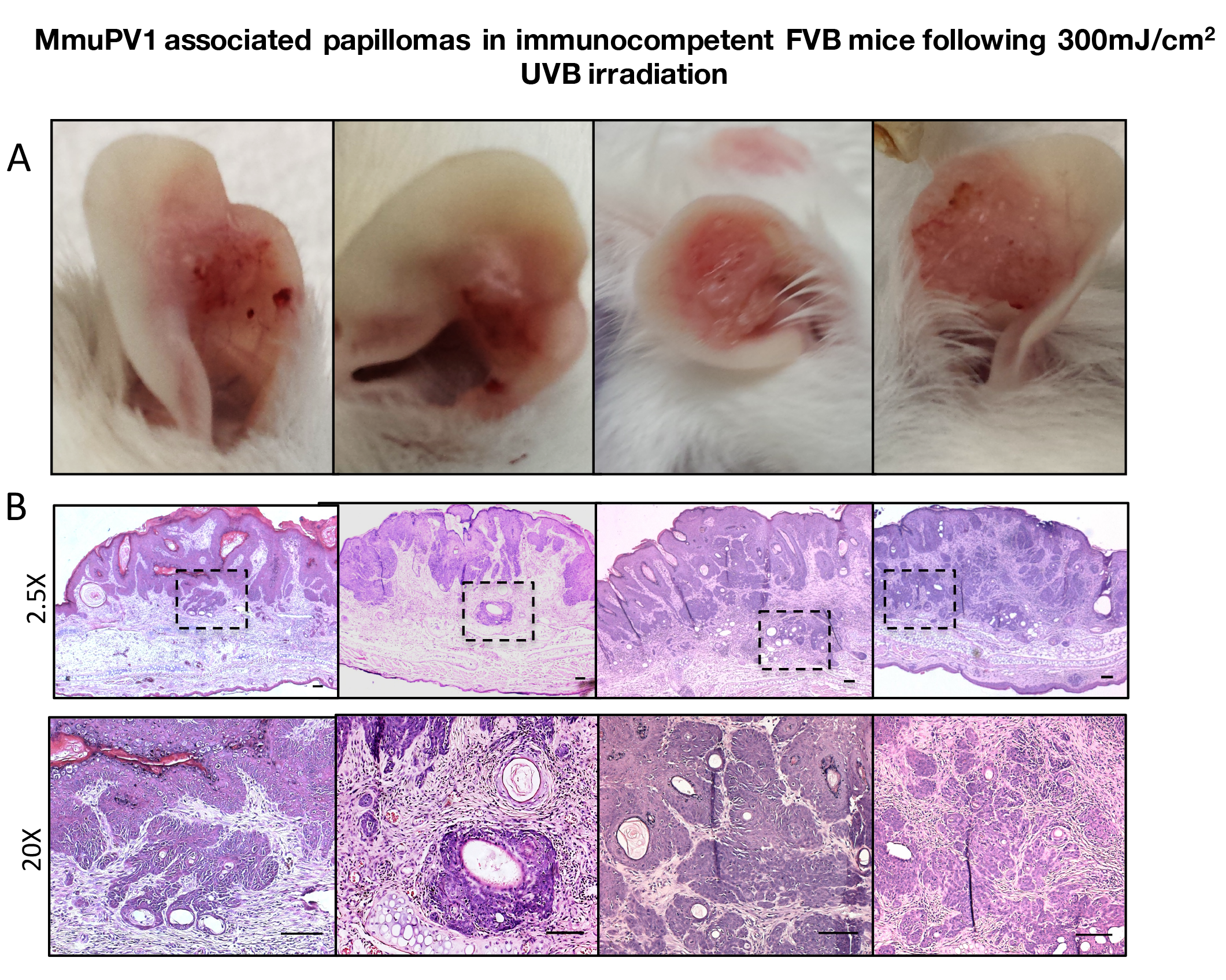

Supplement: S1 Fig — (A) Papillomas on ears of immunocompetent FVB/NJ mice. Examples of papillomas that arose on ears of FVB/NJ mice infected with 108 VGE MmuPV1 followed by irradiation with 300mJ/cm2 UVB. These images represent papillomas at 6 months post infection. (B) Histopathology of MmuPV1-induced ear papillomas in UVB irradiated FVB/NJ mice. Top panel shows H&E images (taken using a 2.5X objective) of lesions. Bottom panel with insets (taken using a 20X objective) indicate corresponding areas of lesions showing focal regions invasivity within the underlying dermis. Scale bars denote 100μm. (TIF) [file ppat.1005664.s001.tif]

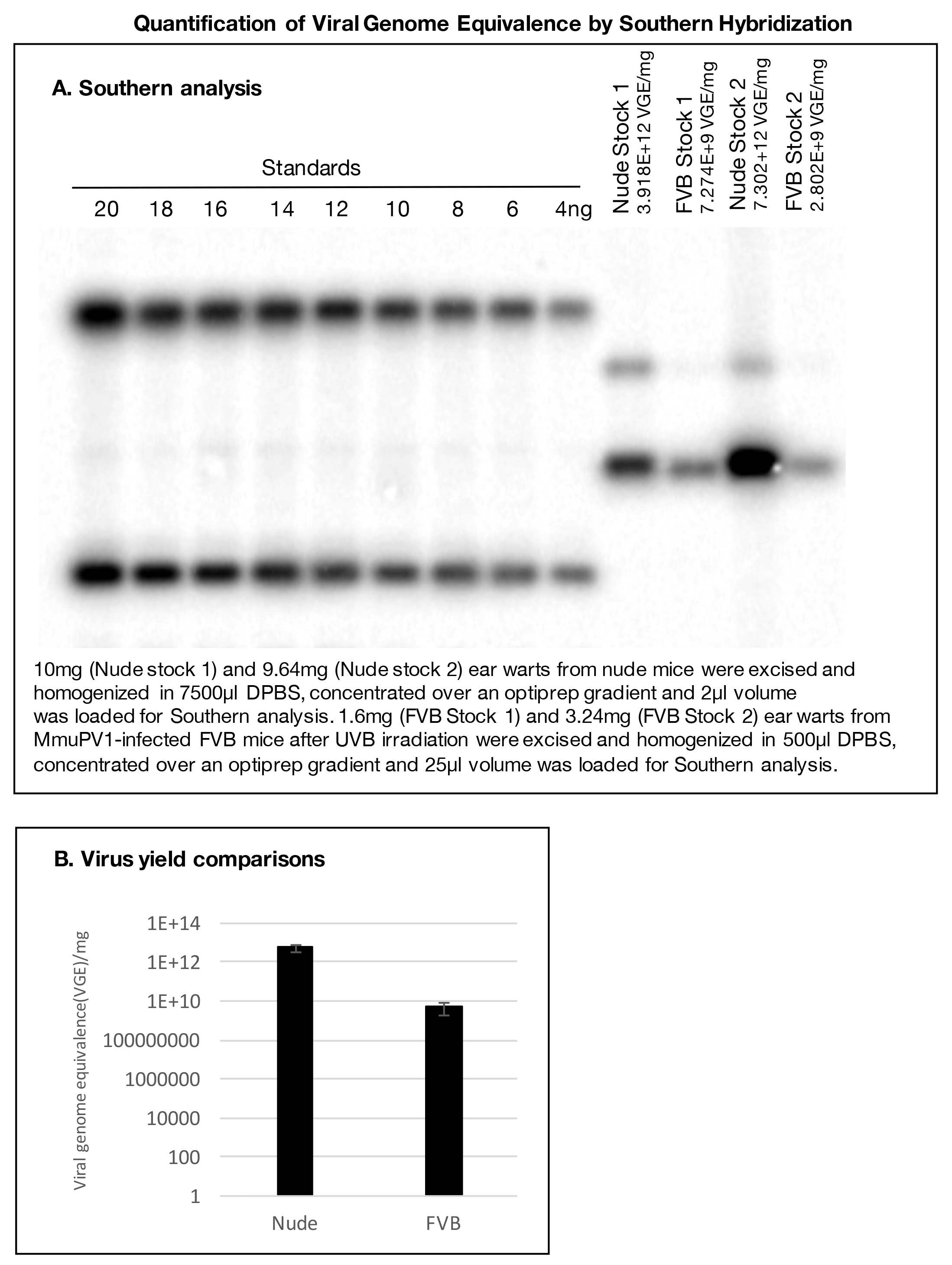

Supplement: S2 Fig — Southern analysis used to quantify virus stocks obtained from MmuPV1 induced ear warts in immunodeficient nude mice and UVB-irradiated FVB/NJ mice. (TIF) [file ppat.1005664.s002.tif]

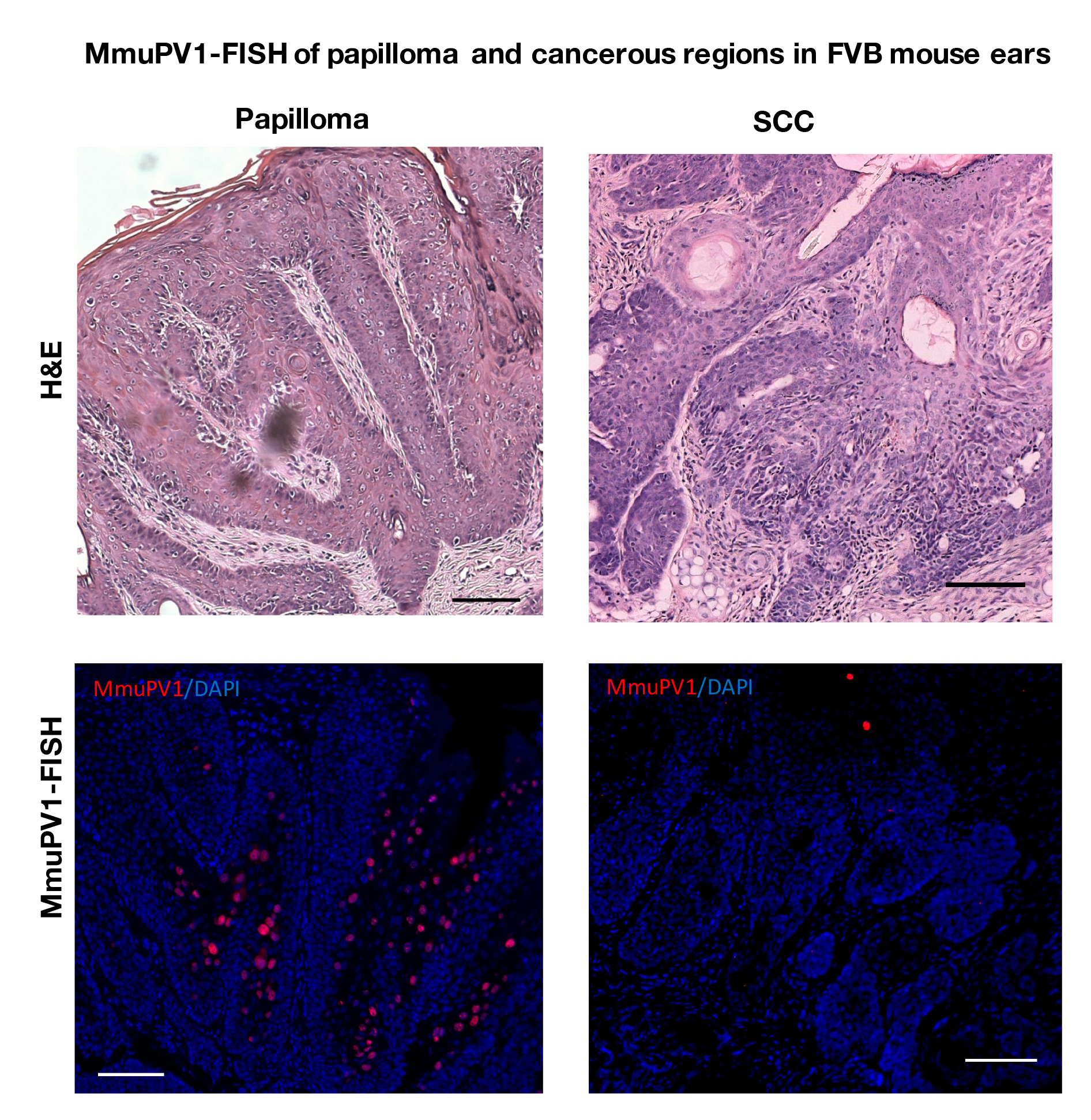

Supplement: S3 Fig — MmuPV1-FISH was performed on papillomas that arose on ears of FVB/NJ mice infected with 108 VGE MmuPV1 followed by irradiation with 300mJ/cm2 UVB. Areas of papillomatosis show presence of amplified viral DNA (red) whereas cancerous regions show little to no presence of amplivied viral DNA. Nuclei were counterstained with DAPI (blue). Scale bars denote 100μm. (TIF) [file ppat.1005664.s003.tif]

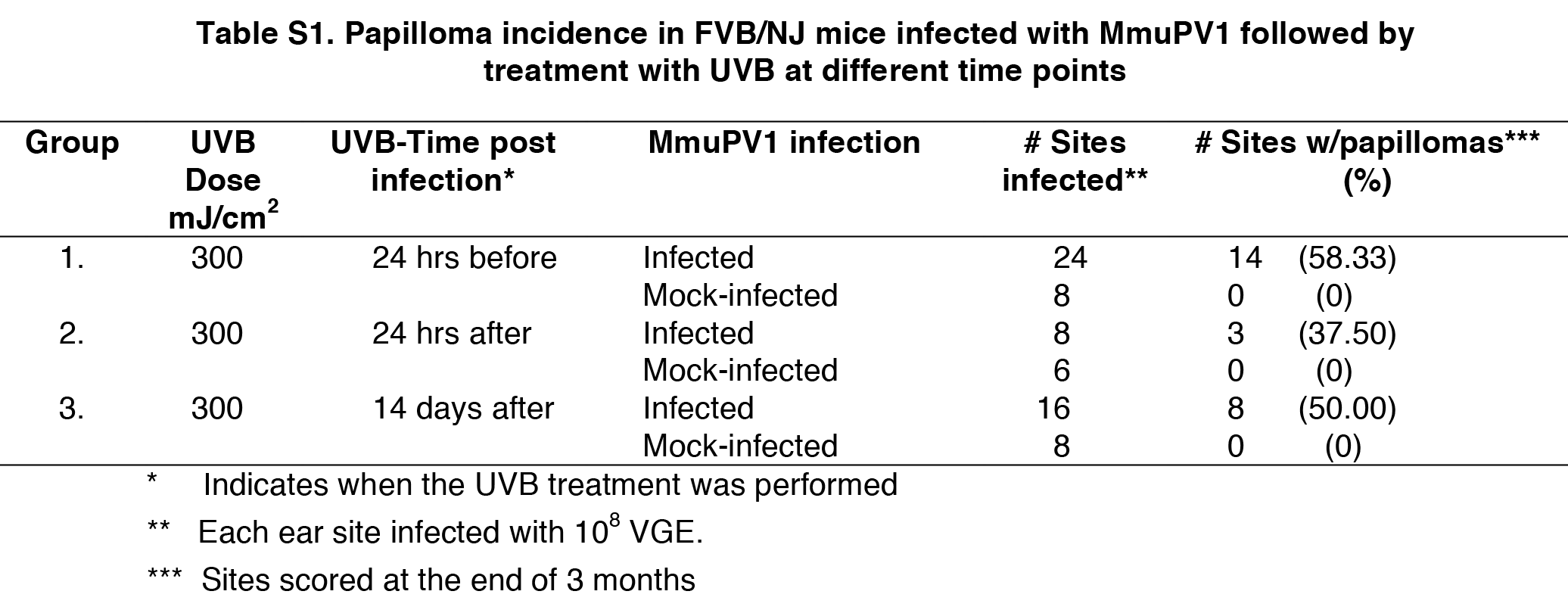

Supplement: S1 Table — FVB/NJ mice were either treated with UVB twenty-four hours prior to infection (Group 1), twenty-four hours post-infection (Group 2) or fourteen days post-infection (Group 3) with MmuPV1. All animals were infected with 108 VGE MmuPV1. UVB dose was 300mJ/cm2. Data shown here represents sites scored at 3 months post-infection. (TIF) [file ppat.1005664.s004.tif]

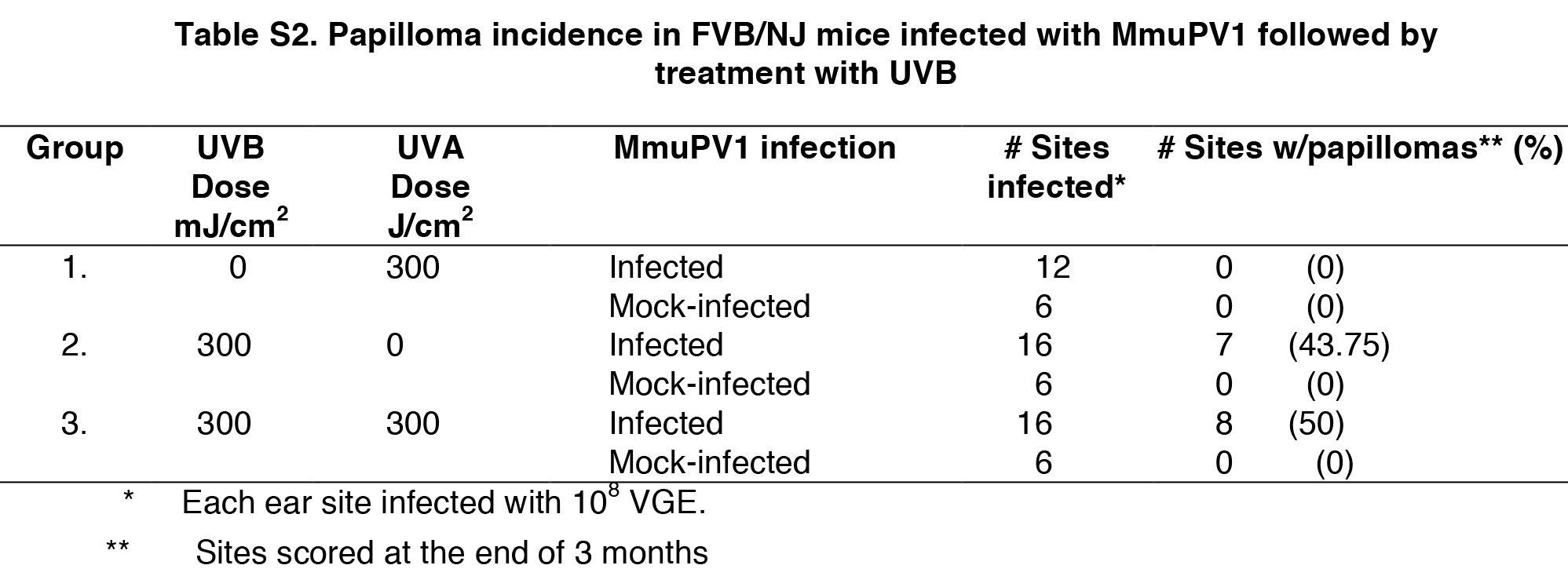

Supplement: S2 Table — FVB/NJ mice were either treated with UVA alone (Group 1), UVB alone (Group 2), or both UVA and UVB (Group 3) twenty-four hours post-infection with 108 VGE MmuPV1. UVB dose was 300mJ/cm2 and UVA dose was 300J/ cm2. Data shown here represents sites scored at 3 months post-infection. (TIF) [file ppat.1005664.s005.tif]

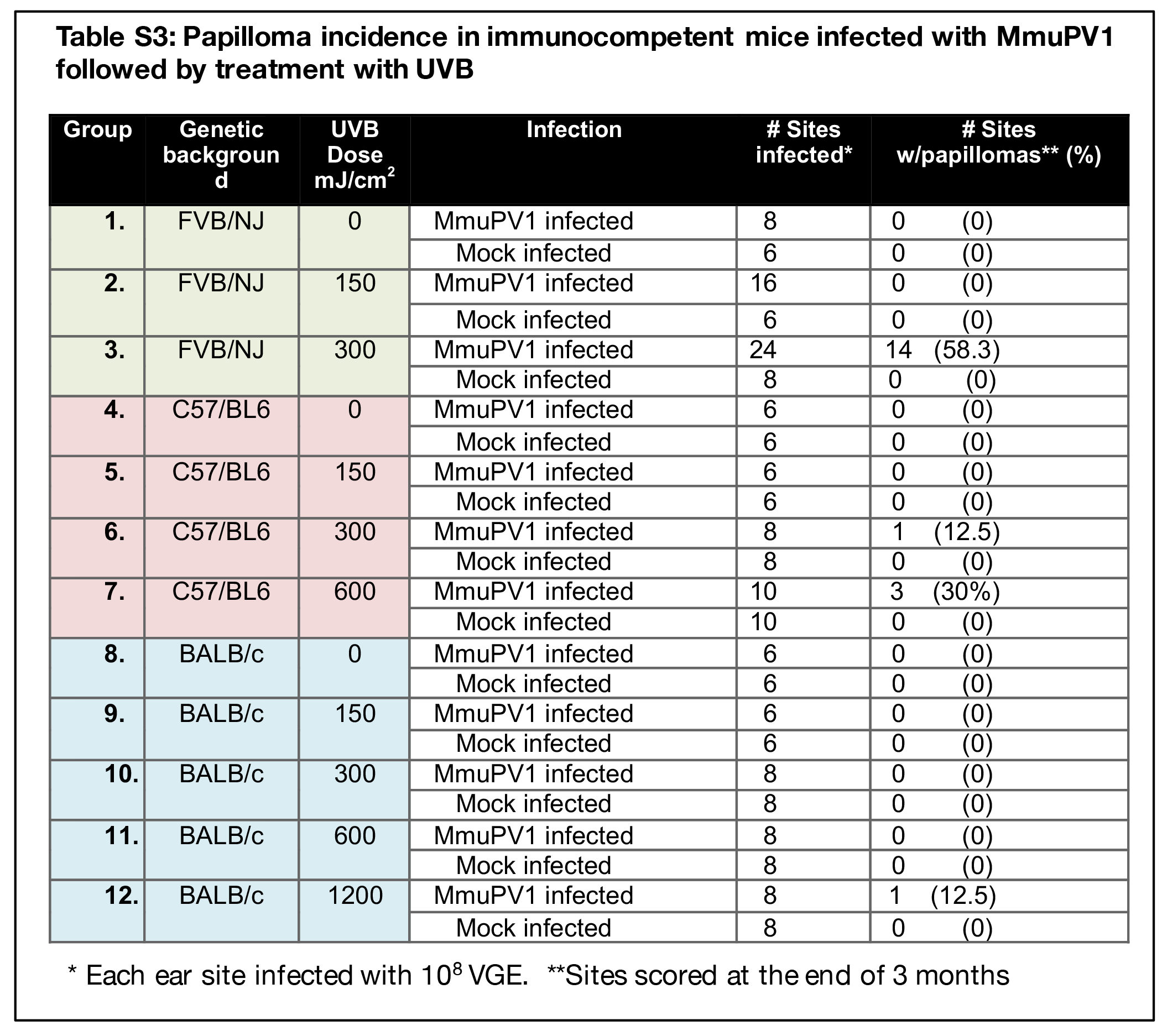

Supplement: S3 Table — Mice of different genetic backgrounds were infected with 108 VGE MmuPV1 and irradiated with designated dose of UVB twenty-four hours post-infection. Data shown here represents sites scored at 3 months post-infection. (TIF) [file ppat.1005664.s006.tif]
