# Supplementary material for: Role of Ultraviolet Radiation in Papillomavirus-Induced Disease
Source: PLoS Pathog. 2016 May 31;12(5):e1005664. doi: 10.1371/journal.ppat.1005664 (PMC4887022; doi:10.1371/journal.ppat.1005664)
Supplement: S1 Text — Text accompanying materials and methods. (PDF) [file ppat.1005664.s007.pdf]

Text S1. Protocol for Southern analysis of MmuPV1.

MmuPV1 plasmid DNA and viral DNA (extracted by denaturation of virions clarified over an optiprep gradient) was separated on a 1X TBE (90 mM Tris, 90 mM boric acid, 2.5 mM EDTA) 0.6% agarose gel (Seakem LE Agarose Lonza Allendale, NJ) at 1 volt/cm for 18-24 hours. The gel was depurinated in 0.25 M HCl for 10 minutes, denatured in two changes of denaturing solution (1.5 M NaCl, 0.5 M NaOH) for 15 minutes each and neutralized in two changes of neutralizing solution (1.5 M NaCl, 1M Tris pH 7.4) for 15 minutes each. DNA was transferred to a positively charged nylon membrane (Hybond N+ Amersham Pittsburgh, PA) using the upward capillary transfer method with 10x SSC (1.5 M NaCl, 150 mM sodium citrate) for 24 hours. The nylon membrane was briefly rinsed in ddH_20 and dried on 3 MM blotting paper (MidSci St. Louis, Mo) for ten minutes before cross-linking with the auto crosslink function of the UV Stratalinker 2400 (Stratagene). After pre-hybridization of the membrane for 15 minutes at 55°C with Church hybridization buffer (250 mM Na_2HPO_4, 1% BSA, 245 mM SDS and 5 mM EDTA pH 8.0), the appropriate radiolabeled MmuPV1 probe was hybridized overnight at 55°C. The membrane was washed five times, five minutes each, with Church wash buffer (140 mM SDS, 80 mM Na_2HPO_4) at 37°C. The hybridized membrane was exposed to a storage phosphor screen (Amersham Pittsburgh, PA) overnight before being scanned with the Typhoon 8610 (Amersham). Brightness and contrast were adjusted using ImageJ version 1.46r to assist with visualization of the printed image. Radiolabeled probes were created by labeling

10 pmoles of oligonucleotides specific for MmuPV1 (synthesized by Integrated DNA Technologies) in the presence of T4 polynucleotide kinase (PNK (New England Biolabs)), 1X T4 PNK buffer (New England Biolabs), 5 mM DTT and 75 µCi of ATP [γ-^{32}P] 6000 Ci/mmol EasyTide (PerkinElmer) at 37°C for 1-2 hours. Unincorporated nucleotides were removed with the micro bio-spin P-30 tris chromatography columns (Bio-Rad Hercules, CA) per the manufacturer's protocol.

Sequences of MmuPV1 oligonucleotides (5'-3'):

1. AACATCGGGTAGGTCAGGCTCAC
2. AGCCCCAAACACAGCTACGACCC
3. AGGCTGCAGCCAGAACTGACTCC
4. ACTTATGCCAGTTGCCGTTCTCA
5. TTGACGCGCCTGCTTCTGTC
6. TCTGTGTTGGAGATTGTGGTGCC
7. TTGCCCCAACCTGCTCACTC
8. ACCCCTTAACCTCCACACTAACCG
9. TGGTGTGTTGGTTTGCTGGGAGATAG
10. ACGAATACGGTTATGGGGGCAC
11. TGGAAATCGGCAAAGGCTACACTC
12. TGAGGCTGAATGTGTGGATGATG
13. AGCACGCTCGCCGCACTTTATT
14. TGCAGGCCTTTGAGTTTCATAAGC
15. TCCCACCGACACCACTGACACA
16. TAAACTCTGCTGGTGGAAGGGTAAC
17. ACACCTGAAGGCCCGTTGC
18. TGCCAGCTGCGGATCTATTCC
19. TGGAGCTCTGGAGAAGGGTGAC
20. TCCCCCACAGCAGTCCATCTC
